# The History of AOSR: Asian Oceanian Society of Radiology

**DOI:** 10.2349/biij.7.4.e30

**Published:** 2011-10-01

**Authors:** Lilian LY Leong, William SC Hare, Kenneth R Thomson

**Affiliations:** 1 Founding President, Hong Kong College of Radiologists, Hong Kong SAR, China; 2 Professor Emeritus of Radiology, The University of Melbourne, Victoria, Australia; 3 Alfred Hospital, Melbourne, Australia

## The Beginnings

In the 1960s, there was a growing awareness of the need to develop closer ties between radiologists in the various nations of Asia and Oceania. Not only would this provide opportunities to learn from one another, but more importantly, it would contribute towards furthering harmonious regional and international relationships. To this end, in the northern hemisphere of this region consideration was given to forming a Far Eastern Society, whilst in the southern hemisphere of the region, national radiological societies in Australia, New Zealand and some South-East Asian countries had similar ideas.

Since 1923 the International Society of Radiology (ISR) had provided leadership as the world authority in radiology by way of the International Congress of Radiology (ICR) held every 4 years, and by establishing important standing committees such as the International Commission on Radiological Protection. With representation from the majority of the world’s nations, ISR was a suitable forum to discuss matters of general interest for members as well as the wider community.

However with the passage of the years, the influence of ISR waned as a result of the emergence of large regional societies. By the mid-1960s a zonal pattern was developing.

In the Americas, the Radiological Society of North America (RSNA), begun in 1928, brought together Canada and the United States and was growing from strength to strength due much to its considerable available financial resources to promote training and research. Another society, the Inter-American Society of Radiology, embracing both North and South America, characterised by being multilingual, had been established in 1933, and continued to the present, but has not reached the level of significance of RSNA. The yearly RSNA congress thus became the main focus for manufacturers and the trade industry to exhibit their latest wares and radiologists from worldwide flocked to Chicago to see what was on offer as well as to attend the excellent academic programmes.

In the European zone, there was the European Association of Radiology (EAR), a federation of national societies founded in 1962, and the European Congress of Radiology (ECR), the organising body of the eponymous congress, which was founded in 1967 and conducted congresses every four years. Over time, no doubt stimulated by the success of RSNA, European radiologists set about to improve the stature of their Society through the European Congress of Radiology. This was well under way by the 1960s and today, ECR is held every year in Vienna.

Another important factor which encouraged the development of regional congresses came from the trade houses. With the exponential escalation of radiology as expected, individual countries, with increasing numbers of radiologists, established their own national annual congresses and looked to the various firms and trade houses for financial and other support. In addition subspecialty societies and meetings evolved, placing further burdens on the resources of companies. Understandably, the commercial world saw that supporting only large regional congresses was a way to rationalise their commitments. By the late 1960s, there was a trend towards the large company headquarters supporting only major congresses, leaving the question of assisting national meetings to their local agents. This was the state of affairs when the thought of setting up a regional society in the Asia and Pacific region was contemplated.

**Figure 1:**
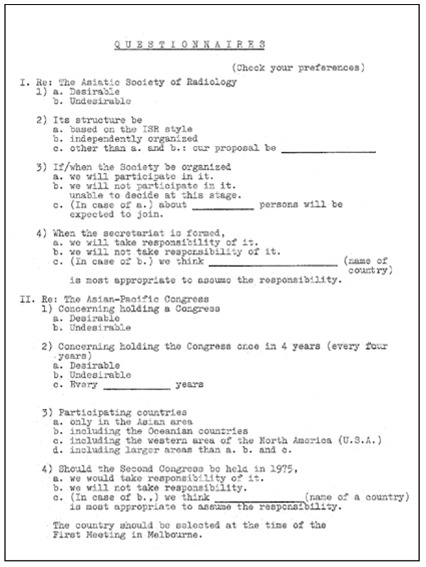
Questionnaires issued by the Preparation Office of the Meeting of Asia Regional Radiologists circulated to 13 constituent nations/territories

**Figure 2:**
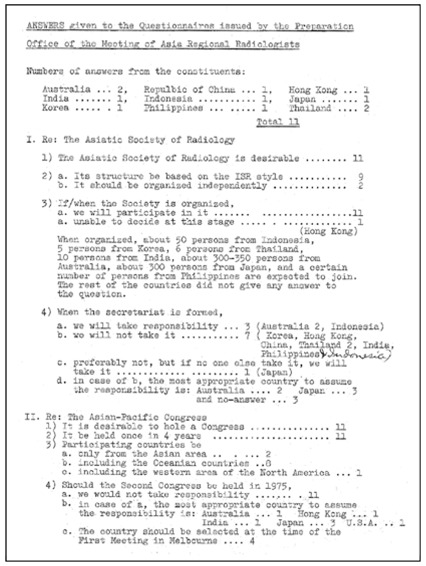
Answers given to the Questionnaires returned to the Preparatory Committee

The idea of setting up the Asian-Oceanian Society of Radiology (AOSR) came about in the late 1960s when questionnaires were circulated to 13 constituent nations/territories and opinions were sought (Fig. 1: Questionnaires issued by the Preparation Office of the Meeting of Asia Regional Radiologists; Fig. 2: Answers given to the Questionnaires). There was a general consensus towards establishing a regional radiological society. In 1969, for the first time the ICR was held in Asia, at the Otani Hotel in Tokyo, Japan, providing the opportunity to discuss the formation of an Asian Oceanian radiological society. This meeting was hosted by the Japanese delegation and was attended by 12 nations (transcribed from original documents), namely, Australia, Ceylon, Hong Kong, India, Indonesia, Japan, Korea, Malaysia, New Zealand, Philippines, Republic of China and Thailand. Incidentally, the ICR in Tokyo was a great success and Professor Tsukimoto was installed as President of ISR and Dr. Adachi as General Secretary of the Congress.

## The Formation of the Society

**Figure 3:**
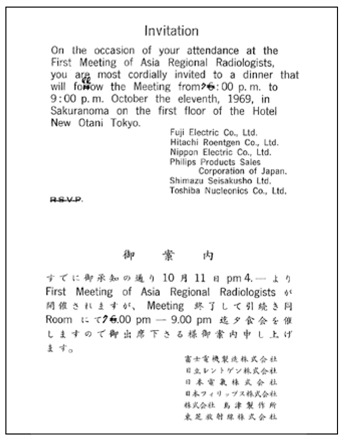
Invitation Card of *The First Meeting of Asian and Oceanian Regional Radiologists*

**Figure 4:**
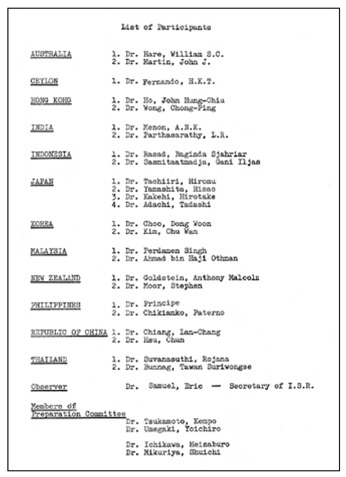
Original List of Participants of *The First Meeting of Asian and Oceanian Regional Radiologists*

**Figure 5:**
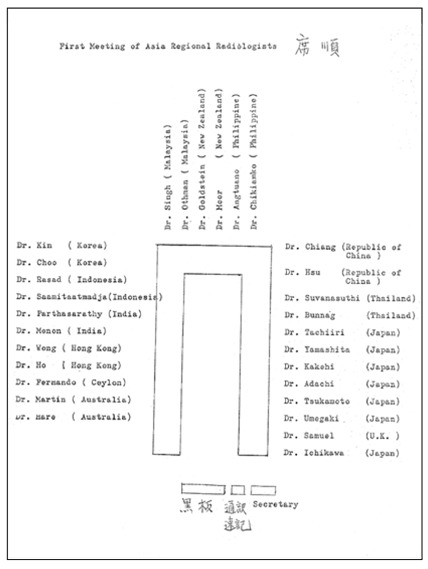
Record of Seating Plan of *The First Meeting of Asian and Oceanian Regional Radiologists*

**Figure 6:**
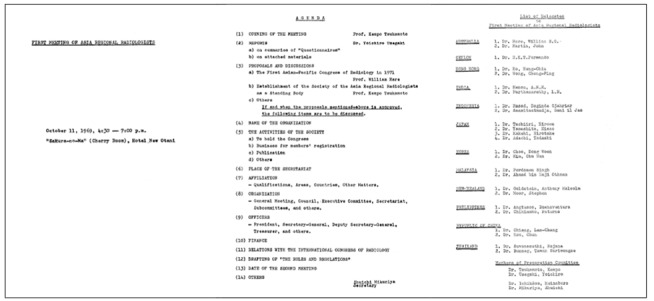
Agenda of The First Meeting of Asian and Oceanian Regional Radiologists on October 11, 1969, 4:30 – 7:00p.m. “Sakura-no-Ma” (Cherry Room), Hotel New Otani, Japan

This first meeting was held during the ICR on 11^th^ October, 1969 at 4:30pm in Hotel New Otani, Tokyo, Japan. Participating delegates, including radiologists and radiotherapists, from the national radiological societies in the region were invited to discuss the formation of a regional society, which would assume the same status as the other two large regional societies, namely the RSNA and the ESR. The meeting was called ***The First Meeting of Asian and Oceanian Regional Radiologists***. There were 30 participants. (Fig. 3: Invitation card; Fig. 4: Original list of participants; Fig. 5: Seating Plan; Fig. 6: Agenda of First Meeting of Asian and Oceanian Regional Radiologists). Professor William Hare was nominated the Chair of the meeting. It was agreed that a society be formed with the boundaries extending from Pakistan in the West to the western shore of the United States of America in the East, and from Korea in the North to Australia and New Zealand in the South. Considerable debate took place on a suitable name for the new Society. Australia and New Zealand had proposed that the society be called “The Asian-Pacific Society of Radiology”, Dr. John Ho of Hong Kong however, pointed out that this was rather restrictive and not sufficiently all-embracing. He suggested that The Asian and Oceanian Society of Radiology would be more appropriate, including, as it does Asia and the islands of the Pacific and Indian Oceans. His recommendation was agreed to, but, as many commented on later, this decision created pronunciation difficulties for years to come, particularly, but not only, for radiologists in non-English speaking countries. It was decided that the English language would be used for all Society’s activities, including the presentation of papers at the proposed congresses to be held every four years.

That meeting also discussed a basic constitution. From the very beginning of this new Society, it was decided that each member nation would be eligible to have two delegates sitting on the AOSR Council, irrespective of the number of members in the national society. For some nations which did not have a constituted association or society, it was agreed that the local radiologists nominate two of their members as delegates to Council. Determining the amount of subscription by each member nation to the Society was complicated by the fact that some member nations only had small number of members. Furthermore, many member nations had diverse financial status. It was agreed to accept the invitation of the Australian Delegates to hold the First Asian-Oceanian Congress in Melbourne, Australia, in 1971. Professor William S.C. Hare was elected the Foundation President of the Society and Dr. John J. Martin the first Honorary Secretary/Treasurer.

The nations forming the Society initially were: Korea, Japan, Taiwan, Hong Kong, Cambodia, Malaysia, Singapore, Thailand, India, Bangladesh, Sri Lanka, Indonesia, Philippines, Hawaii, Western Samoa, Fiji, New Zealand, Australia and Pakistan.

## Statutes for the Asian and Oceanian Society of Radiology

**Figure 7:**
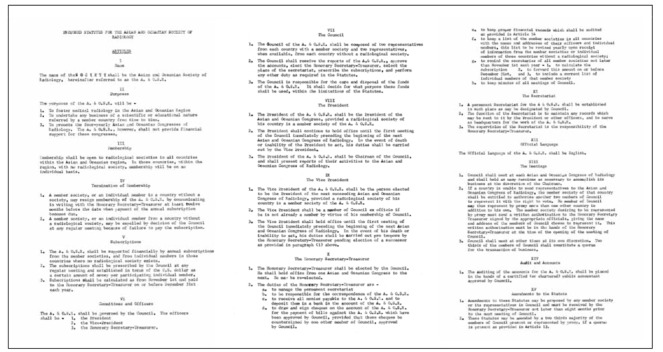
Proposed Statutes for the Asian and Oceanian Society of Radiology

**Figure 8:**
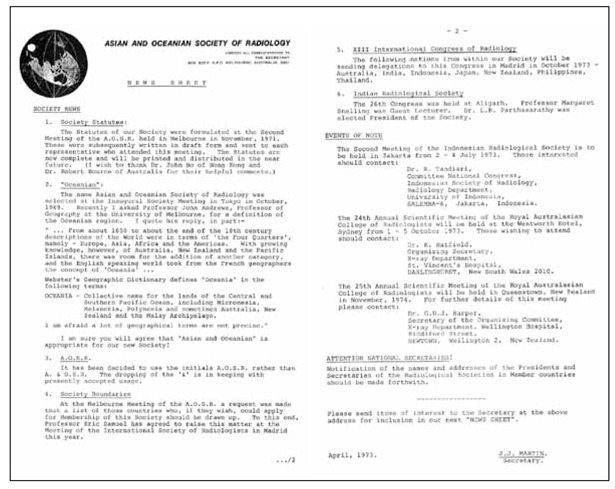
News Sheet, April 1973 - 1^st^ Communication Paper with Member Societies

**Figure 9:**
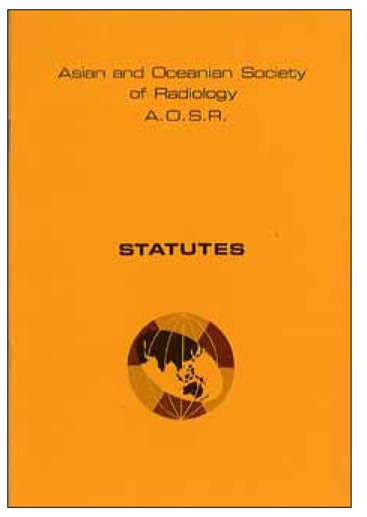
Asian and Oceanian Society of Radiology A.O.S.R. STATUTES printed in 1993

The proposed Statutes for the Asian and Oceanian Society of Radiology were formulated by Dr. J.J. Martin, Dr. Robert Bourne of Australia and Dr. John Ho of Hong Kong (Fig. 7: Proposed Statutes; Fig. 8: News Sheet, April 1973) at the Second Meeting of the AOSR in Melbourne and was sent to all members. The printed Statues was distributed in 1993 (Fig. 9: Asian and Oceanian Society of Radiology Statutes).

## Executive Committee and Executive Council of AOSR

***Executive Committee of AOSR*:** The members of the Executive Committee were nominated and voted in by the Member Societies. The Executive Committee in the beginning consisted only of a few members. At the AOSR Council meeting in Kuala Lumpur in 1995, it was decided and endorsed by the Executive Committee to increase the membership of the Executive Committee by three, with arrangements to meet between AOCRs.

***Executive Council of AOSR*:** The Executive Council presently consists of the President, President-elect, Secretary, Treasurer, four Ordinary Executive Council Members, Immediate Past-President and Co-opted Councillor with 7 Committees. For the General Assembly, it normally conducts the Assembly meeting during every AOCR and is made up of the Executive Council and the representatives from Member Societies.

## Registration and Constitutions

As AOSR grew, there came the need for this Society to be formally registered and to have a proper Society Constitution. The Society was first registered in Singapore. However, due to various regulations, including taxation required of registered Societies in Singapore, it was decided to move the site of AOSR registration. It was first relocated to Australia in 2005 and managed by The Royal Australian and New Zealand College of Radiologists (RANZCR). Then in 2010, Korean Society of Radiology (KSR) took over the management and AOSR was registered in Korea.

## Presidents of Asian Oceanian Society of Radiology (Fig. 10)

**Figure 10:**
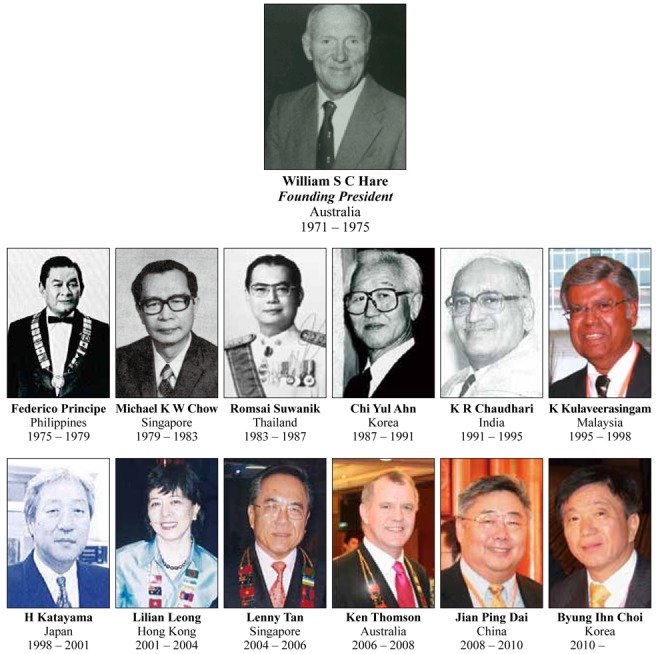
Presidents of Asian Oceanian Society of Radiology

## AOSR President’s Chain of Office

**Figure 11:**
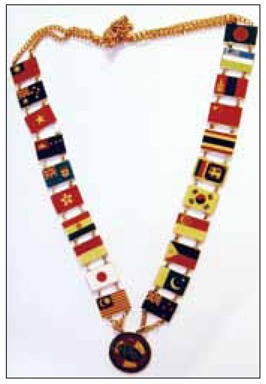
AOSR President’s Chain of Office

The original chain was donated by Dr. Principe of Philippine, but regrettably, it later become lost. It was remade and was donated by Korean Radiological Society under the leadership of Professor Man Chung Han (Fig. 11: AOSR President’s Chain of Office).

## AOSR Gold Medallists

**Table:** 

**William SC Hare**,	Australia	(2001)
**Man-Chung Han**,	Korea	(2001)
**K Kulaveerasingam**,	Malaysia	(2001)
**Holger Peterrson**,	Sweden	(2001)
**Charles A Gooding**,	USA	(2004)
**Hitoshi Katayama**,	Japan	(2004)
**Guozhen Li**,	China	(2006)
**Anne G Osborn**,	USA	(2006)
**Lilian Leong**,	Hong Kong	(2008)
**Joseph KT Lee**,	USA	(2008)
**Alexander R Margulis**,	USA	(2010)
**Sudarshan Aggarwal**,	India	(2010)

## Activities of AOSR

**Figure 12:**
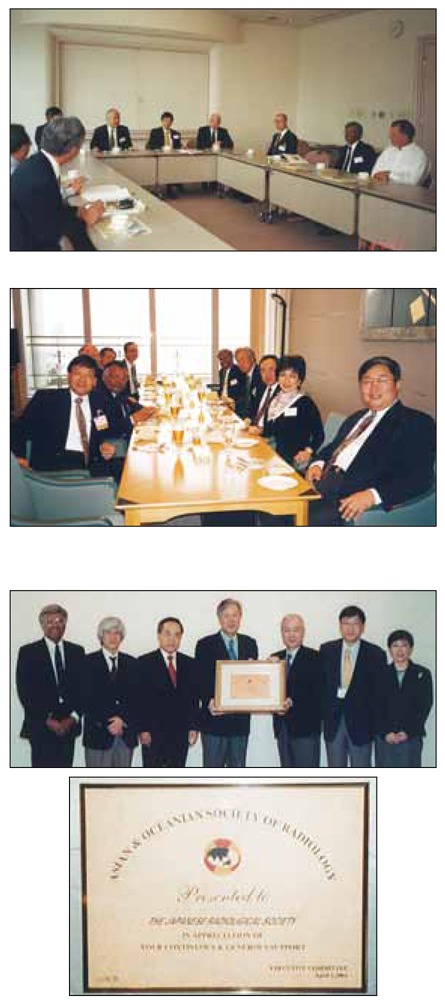
AOSR Executive Committee Meetings

Though AOSR does not possess a permanent office address, the Executive Committee of AOSR can regularly conduct her business meetings during each AOCR and through support of her Member Societies. JCMP and Japan Radiological Society had been inviting members of Executive Committee to attend their annual congress for such purposes (Fig. 12: AOSR Executive Committee Meetings). Other Societies like Chinese Radiological Society, Hong Kong College of Radiologists, etc. had done likewise.

## Satyapal Aggarwal Memorial Lecture

Through Dr. Sudarshan Aggarwal, a donation was made and contributed to the setting up of Satyapal Aggarwal Lecture in memory of his brother, Dr. Satya Pal Aggarwal, the renowned Indian radiologist who had been the President of the Indian Radiological Association and installed the first CT in Delhi. The donation contributed to the invitation of international eminent radiologists to deliver the Satyapal Aggarwal Lecture which was always a highlight of the AOCR. The inaugural Aggarwal Lecture was delivered by Professor William Hare in 1991, followed by Prof. Alexander Margulis, Prof. Roberto Passariello, Dr. Nicholas Gourtsoyiannis, Prof. Philippe Grenier, Prof. Hedvig Hricak and Prof. William Bradley.

## AOCR : Asian Oceanian Congress of Radiology

**Figure 13:**
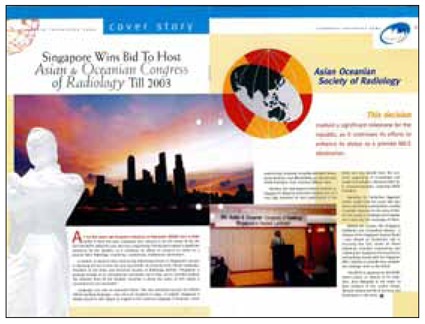
Singapore Convention News, Second Issue 1998 - Singapore was accepted as the semipermanent venue for the *9^th^ & 10^th^ Asian and Oceanian Congress of Radiology in 2001 & 2004*

At the beginning, the AOCR took place every four years. Then the interval was shortened to every 3 years. At the 8^th^ AOCR in Kobe, Japan in 1998, it was resolved to organise the AOCR every 2 years. There was also considerable discussion on establishing a permanent venue for AOCR, using RSNA and ECR as examples. Singapore had bidded to become the permanent congress venue. The proposal was considered and Singapore was accepted as the semipermanent venue for the next 2 Congresses (Fig. 13: Singapore Convention News, Second Issue 1998). The decision of permanent/ semi-permanent venue was to be reviewed after that. In the 10^th^ AOCR in 2003, after the review, it was decided that it was in the interest of the region to rotate the venues of AOCR, thus the bidding for organising AOCR by Member Societies remains. In the past 40 years, there have been thirteen successful AOCR organised due to the enthusiasm and hard work of Member Societies and their dedicated radiologists. Though attendance varied, affected by situation like the SARS outbreak, economic fluctuation, and post-earthquake sentiments, the Congresses were always successful in providing a good forum for academic and cultural exchange. It remains as the most important radiological meeting of the region, well supported by the profession and the industry. With sponsorships for young radiologists, AOCR has been providing many learning opportunities for our radiologists in training.

## AOCR Venues

**Table:** 

1^st^ AOCR	**Melbourne, Australia**	**1971**
2^nd^ AOCR	**Manila, Philippines**	**1975**
3^rd^ AOCR	**Singapore**	**1979**
4^th^ AOCR	**Bangkok, Thailand**	**1983**
5^th^ AOCR	**Seoul, Korea**	**1987**
6^th^ AOCR	**New Delhi, India**	**1991**
7^th^ AOCR	**Kuala Lumpur, Malaysia**	**1995**
8^th^ AOCR	**Kobe, Japan**	**1998**
9^th^ AOCR	**Singapore**	**2001**
10^th^ AOCR	**Singapore**	**2003**
11^th^ AOCR	**Hong Kong**	**2006**
12^th^ AOCR	**Seoul, Korea**	**2008**
13^th^ AOCR	**Taipei, Chinese Taipei**	**2010**
14^th^ AOCR	**Sydney, Australia**	**2012**

## Picture Gallery of Asian Oceanian Congress of Radiology (AOCR)

**Figure:**
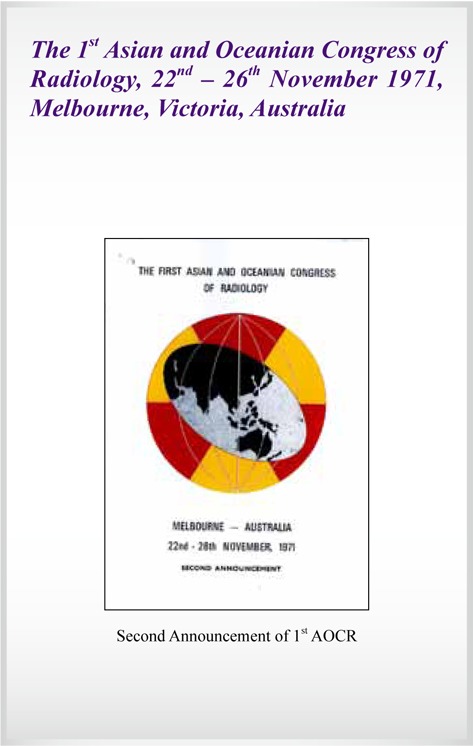


**Figure:**
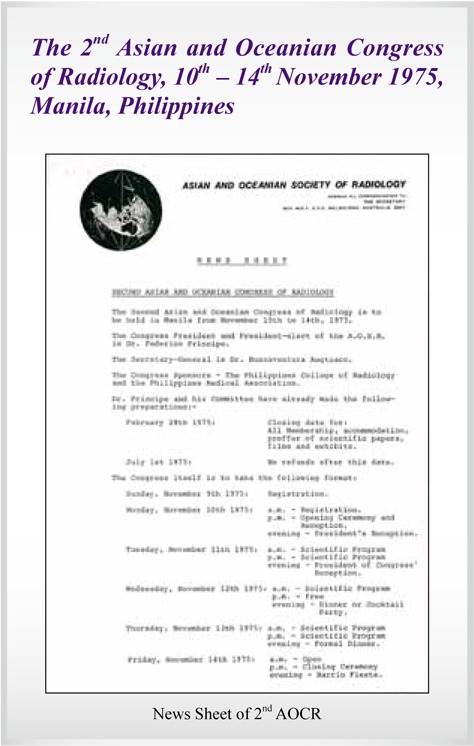


**Figure:**
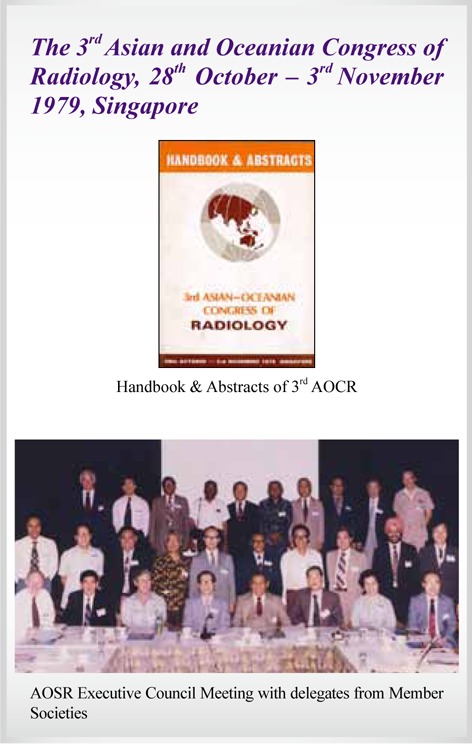


**Figure:**
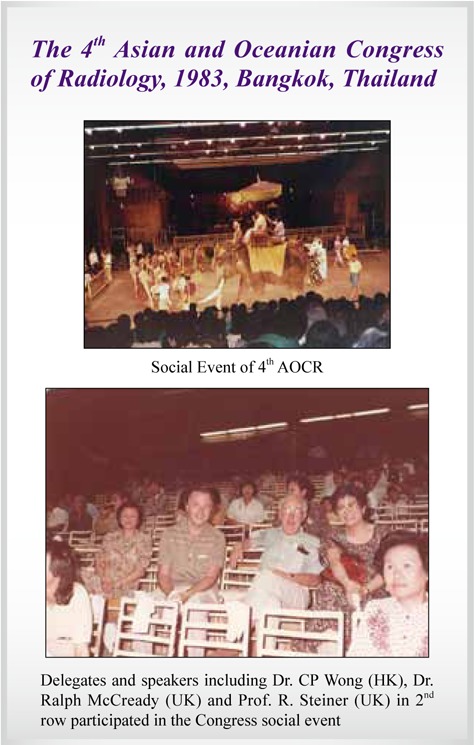


**Figure:**
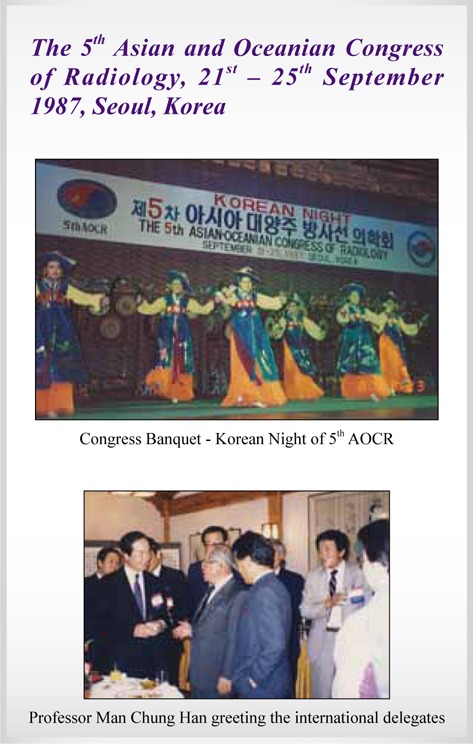


**Figure:**
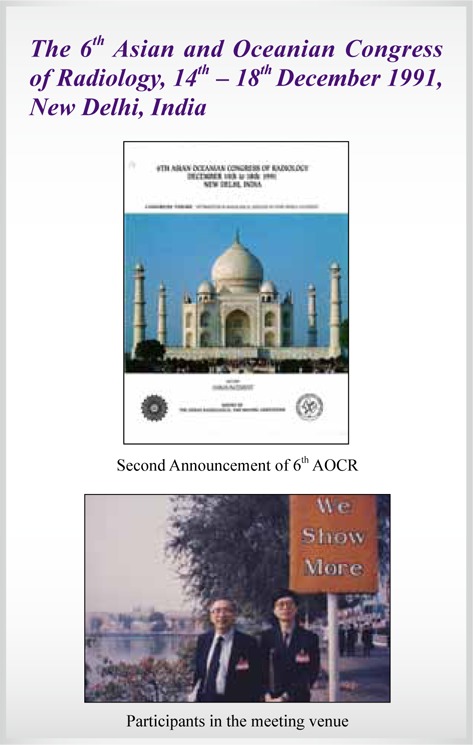


**Figure:**
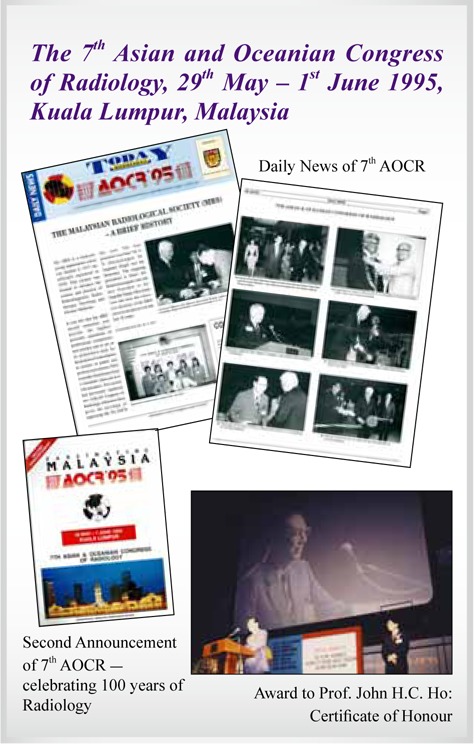


**Figure:**
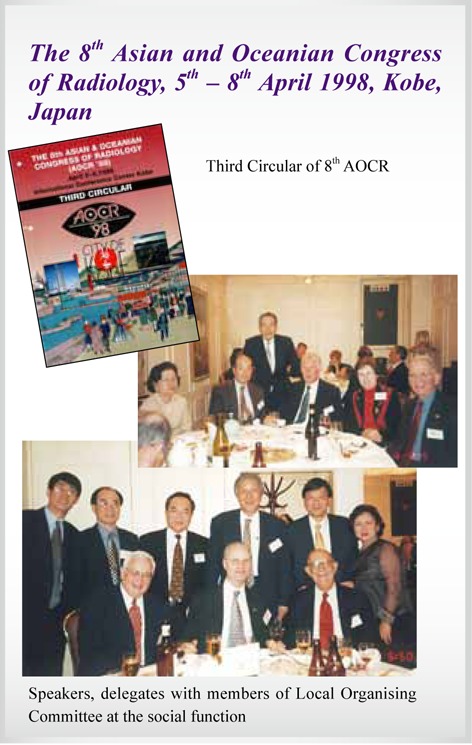


**Figure:**
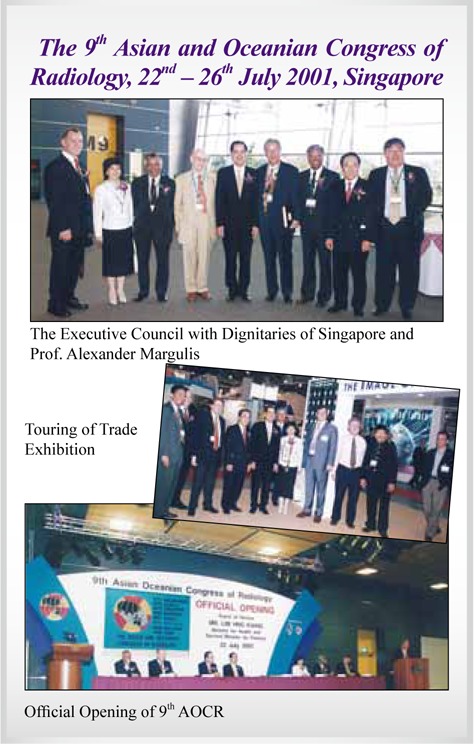


**Figure:**
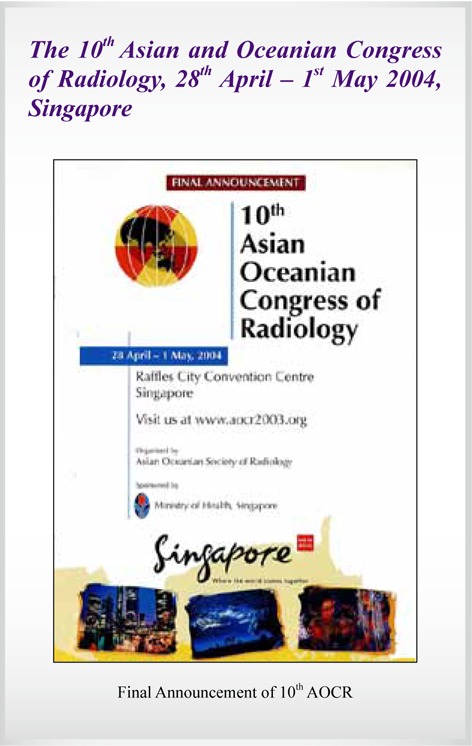


**Figure:**
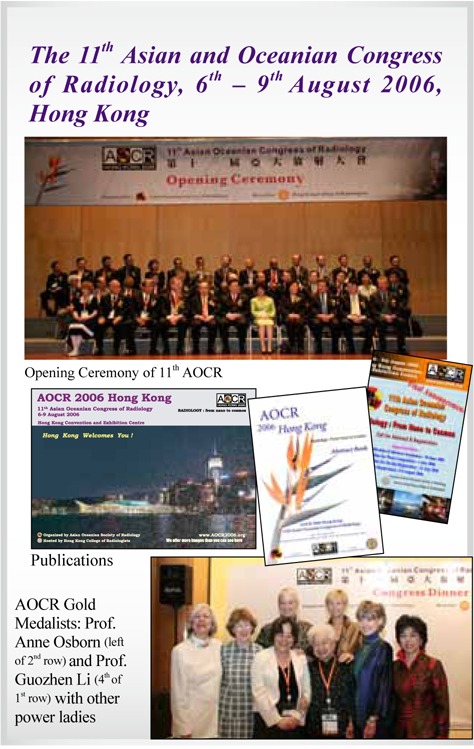


**Figure:**
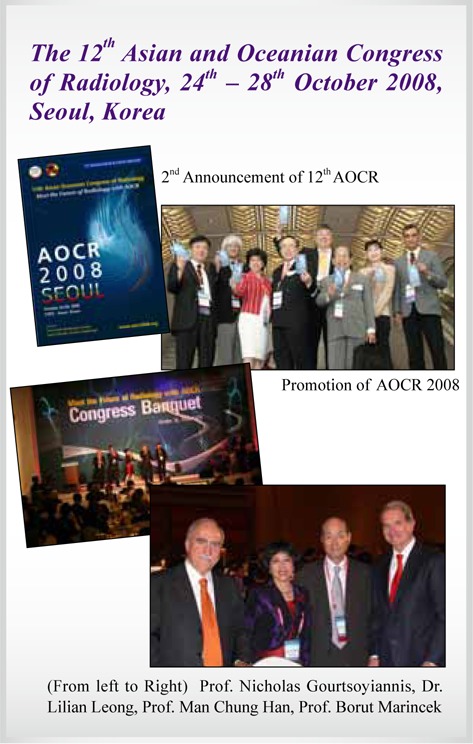


**Figure:**
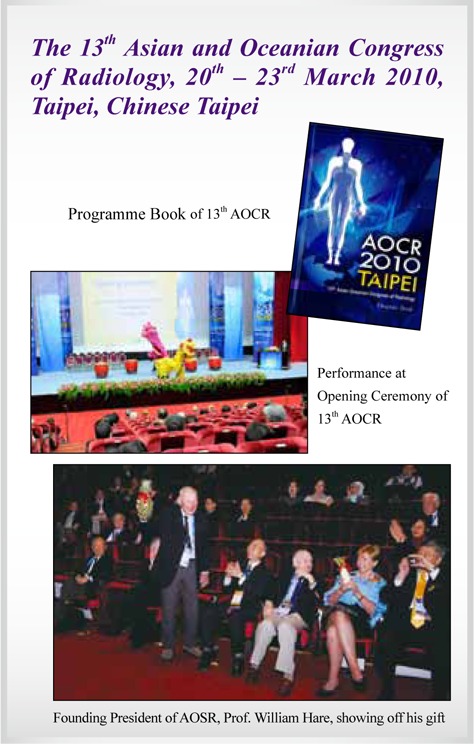


**Figure:**
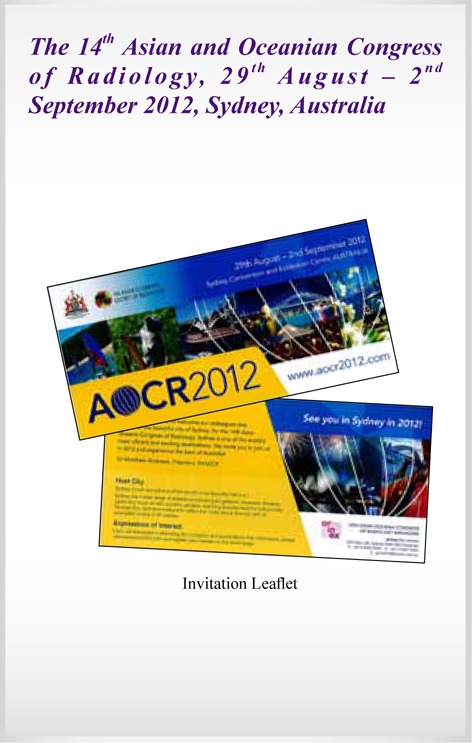


## Publications and Educational Activities

**Figure 14:**
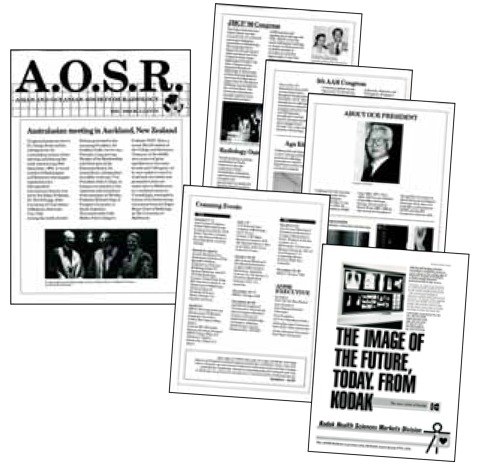
A.O.S.R. December 1988 Bulletin

**Figure 15:**
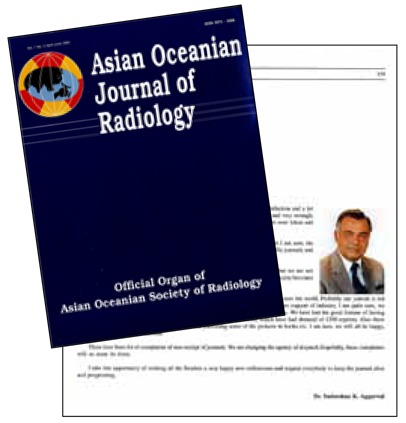
Asian Oceanian Journal of Radiology and Chief Editor, Dr. Sudarshan K. Aggarwal

Much efforts were made in early days to keep good communications between Member Societies and their members. The earliest attempt (1973) was the News Sheet, which was mailed to contacts of National Societies (same Fig. 8. referred). With the support of a sponsor, Prof. Hare started the publication of the AOSR Bulletin in 1979 (Fig. 14: AOSR December 1988 Bulletin). That served as a useful tool to keep the profession in the region updated of the happenings. Then Dr. Sudarshan K. Aggarwal of India managed to get funding to start the AOSR Journal in 1995, he himself being the Editor-in-Chief (Fig. 15: Asian Oceanian Journal of Radiology). The Journal had attracted many good contributions from the region. However, regrettably, when sponsorship ceased, both publications had to be discontinued.

**Figure 16:**
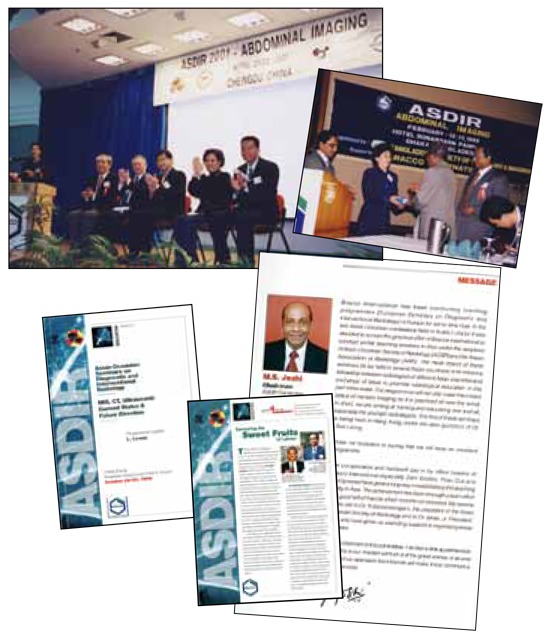
Asian-Oceanian Seminars on Diagnostic and Interventional Radiology

From 1996, through the hard work of Dr. K. Kulaveerasingam, the 7^th^ President of AOSR, and Dr. M.S. Joshi, the Chairman of the ASDIR Committee, and with the sponsorship of Bracco International, there were also very successful series of ASDIR, Asian-Oceanian Seminars on Diagnostic and Interventional Radiology under the umbrella of AOSR and Asean Association of Radiology (AAR). These seminars were held in different countries to allow more area coverage and to enhance fellowship between Asian radiologists. To further promote radiological education in the region, the teachers and speakers of ASDIR were nominated from the local organisers and from the region (Fig. 16).

AOSR also forms an important medium for conducting regional research and surveys. There have been useful surveys into manpower analysis, teleradiology development, etc. in different countries. In 1998, a Committee on Training, Training Centres and Certification was set up under AOSR Education Workshop with the aim of providing an advisory role on training standards, training guidelines, and to facilitate training information, resources on teaching materials, and opportunities for training sponsorship. A survey was conducted on the training centres in Bangladesh, China, Chinese Taipei, Hong Kong China, Indonesia, Japan, Korea, Malaysia, Philippines, Singapore, Sri Lanka and Thailand. The report was released in 1999. The Committee was chaired by Dr. FL Chan (Hong Kong) and members were SK Aggarwal (India), KS Cho (Korea), HM Liu (Chinese Taipei) and S Suthipongchai (Thailand). AOSR has always been active in educational activities and continues to be so, represented in various forums organised by RSNA, ISR/ICR, International Radiology Quality Network (IRQN) and sharing its experience and progress with other regional radiological organisations.

## Present

This year marks the 40^th^ year of Anniversary of AOSR. There are 23 Member Societies comprising of Bangladesh Society of Radiology & Imaging, Chinese Society of Radiology, Chinese Taipei Society of Radiology, College of Radiology Academy of Medicine of Malaysia, Fiji Radiology, Hong Kong College of Radiologists, Indian Radiological & Imaging Association, Indonesian Radiological Society, Japan Radiological Society, Korean Society of Radiology, Macau Radiology Association, Mongolian Radiological Society, Nepal Radiologists' Association, Philippine College of Radiology, Radiological Society of Pakistan, Radiologist Ministry of Health Tonga, Royal College of Radiology of Thailand, Samoa Society of Radiology, Singapore Radiological Society, Sri Lanka College of Radiologists, The Royal Australian & New Zealand College of Radiologists, Uzbekistan Radiology Society and Vietnam Society of Radiology and Nuclear Medicine. AOSR is now registered in Korea and managed by the Korean Society of Radiology. The website is http://www.theaosr.org. The Asian Oceanian Journal of Radiology (AOJR) is affiliated with the Biomedical Imaging & Intervention Journal (BIIJ).

With the support of the Member Societies and healthcare professionals, one can anticipate the continual growth of AOSR with the rapid advancement of radiology in our region.

